# Data-Driven Two-Stage Framework for Identification and Characterization of Different Antibiotic-Resistant Escherichia coli Isolates Based on Mass Spectrometry Data

**DOI:** 10.1128/spectrum.03479-22

**Published:** 2023-04-12

**Authors:** Chia-Ru Chung, Hsin-Yao Wang, Chun-Han Yao, Li-Ching Wu, Jang-Jih Lu, Jorng-Tzong Horng, Tzong-Yi Lee

**Affiliations:** a Department of Computer Science and Information Engineering, National Central University, Taoyuan, Taiwan; b Department of Laboratory Medicine, Chang Gung Memorial Hospital at Linkou, Taoyuan, Taiwan; c Ph.D. Program in Biomedical Engineering, Chang Gung University, Taoyuan, Taiwan; d Department of Biomedical Sciences and Engineering, National Central University, Taoyuan, Taiwan; e College of Medicine, Chang Gung University, Taoyuan, Taiwan; f Department of Medical Biotechnology and Laboratory Science, Chang Gung University, Taoyuan, Taiwan; g Department of Bioinformatics and Medical Engineering, Asia University, Taichung, Taiwan; h Institute of Bioinformatics and Systems Biology, National Yang Ming Chiao Tung University, Hsinchu, Taiwan; American Type Culture Collection

**Keywords:** fluoroquinolones, cephalosporins, penicillins, matrix-assisted laser desorption ionization–time of flight mass spectrometry, MALDI-TOF MS, machine learning, antimicrobial resistance, cephalosporin, penicillin

## Abstract

In clinical microbiology, matrix-assisted laser desorption ionization–time-of-flight mass spectrometry (MALDI-TOF MS) is frequently employed for rapid microbial identification. However, rapid identification of antimicrobial resistance (AMR) in Escherichia coli based on a large amount of MALDI-TOF MS data has not yet been reported. This may be because building a prediction model to cover all E. coli isolates would be challenging given the high diversity of the E. coli population. This study aimed to develop a MALDI-TOF MS-based, data-driven, two-stage framework for characterizing different AMRs in E. coli. Specifically, amoxicillin (AMC), ceftazidime (CAZ), ciprofloxacin (CIP), ceftriaxone (CRO), and cefuroxime (CXM) were used. In the first stage, we split the data into two groups based on informative peaks according to the importance of the random forest. In the second stage, prediction models were constructed using four different machine learning algorithms−logistic regression, support vector machine, random forest, and extreme gradient boosting (XGBoost). The findings demonstrate that XGBoost outperformed the other four machine learning models. The values of the area under the receiver operating characteristic curve were 0.62, 0.72, 0.87, 0.72, and 0.72 for AMC, CAZ, CIP, CRO, and CXM, respectively. This implies that a data-driven, two-stage framework could improve accuracy by approximately 2.8%. As a result, we developed AMR prediction models for E. coli using a data-driven two-stage framework, which is promising for assisting physicians in making decisions. Further, the analysis of informative peaks in future studies could potentially reveal new insights.

**IMPORTANCE** Based on a large amount of matrix-assisted laser desorption ionization–time-of-flight mass spectrometry (MALDI-TOF MS) clinical data, comprising 37,918 Escherichia coli isolates, a data-driven two-stage framework was established to evaluate the antimicrobial resistance of E. coli. Five antibiotics, including amoxicillin (AMC), ceftazidime (CAZ), ciprofloxacin (CIP), ceftriaxone (CRO), and cefuroxime (CXM), were considered for the two-stage model training, and the values of the area under the receiver operating characteristic curve (AUC) were 0.62 for AMC, 0.72 for CAZ, 0.87 for CIP, 0.72 for CRO, and 0.72 for CXM. Further investigations revealed that the informative peak *m/z* 9714 appeared with some important peaks at *m/z* 6809, *m/z* 7650, *m/z* 10534, and *m/z* 11783 for CIP and at *m/z* 6809, *m/z* 10475, and *m/z* 8447 for CAZ, CRO, and CXM. This framework has the potential to improve the accuracy by approximately 2.8%, indicating a promising potential for further research.

## INTRODUCTION

The abuse of antibiotics has accelerated the emergence of antibiotic-resistant microorganisms. Antibiotic abuse or overuse, in particular, induces the artificial selection of bacteria and accelerates the evolutionary process to the detriment of all individuals (1). An increasing number of patients infected by antibiotic-resistant bacteria do not receive timely treatment because of the lengthy turnaround time for testing antimicrobial resistance (AMR). Furthermore, overcoming the resistance of Gram-negative bacteria to several routinely used antibiotics is challenging (2). Because Gram-negative bacteria have inner cellular membranes and outer membranes containing lipopolysaccharides, it is difficult for small molecules to pass through the outer membrane at a sufficient concentration level through narrow porins (3). Escherichi0a coli is a common Gram-negative bacterium. Highly unstable and genetically diverse E. coli populations have also been found (4). It generates β-lactamase and extended-spectrum β-lactamases (ESBLs), which confer antibiotic resistance to cefotaxime and ceftazidime (5). In conclusion, gaining a better knowledge of antibiotic-resistant E. coli and its treatment is important but challenging.

Several antibiotics, such as ciprofloxacin (CIP), ceftazidime (CAZ), ceftriaxone (CRO), cefuroxime (CXM), and amoxicillin (AMC), are effective against E. coli infections. CIP, a second-generation fluoroquinolone, is effective against most Gram-negative bacteria and moderately effective against Gram-positive bacteria. CIP inhibits DNA gyrase in bacteria, which is the primary mode of action of quinolones (6, 7), making it effective against β-lactamase-producing bacteria (8). As a result, it has been widely used for Gram-negative bacteria and is effective against large groups of bacteria. CAZ, CRO, and CXM are cephalosporins of various generations. Cephalosporins and AMC inhibit bacterial cell wall synthesis, rendering the cells mechanically weak (8).

Matrix-assisted laser desorption ionization–time-of-flight (MALDI-TOF) mass spectrometry (MS) is widely used in clinical microbiology laboratories for bacterial species identification (9–13). Compared with molecular biology and conventional biochemical techniques, MALDI-TOF MS is a rapid, cost-effective, and reliable technology for identifying bacterial species. MALDI-TOF MS mass spectra can represent a whole bacterial cell based on the composition of numerous proteins (14). The identified protein expression patterns can be further analyzed and extended to the protein database (10). Furthermore, antibiotic resistance strains can also be detected using MALDI-TOF MS, such as methicillin-resistant Staphylococcus aureus (MRSA) (15–18), vancomycin-resistant enterococci (19, 20), and Bacteroides fragilis (21). However, detecting AMR in *Enterobacteriaceae* remains challenging. In addition, the completeness of the data sets and the population of bacteria have a significant impact on the performance of these systems (13).

Several studies have reported the rapid detection of AMR in E. coli based on the MALDI Biotyper antibiotic susceptibility test rapid assay (MBT-ASTRA) (Table 1) (22–24). Based on 14 E. coli isolates, Jung et al. obtained only one misclassification for ciprofloxacin nonsusceptibility and five for piperacillin-tazobactam using MBT-ASTRA (22). Furthermore, Sauget et al. used MBT-ASTRA to evaluate 103 E. coli-positive blood cultures and obtained 95% and 84% accuracy in the rapid identification of AMRs for amoxicillin and cefotaxime, respectively (23). De Carolis et al. sought to detect cefotaxime-resistant E. coli and Klebsiella pneumoniae using the MALDI-TOF spectrum and the presence or absence of peaks associated with cefotaxime (24). Although MBT-ASTRA and comparable methods are relatively accurate, preincubation requires additional time. Rapid detection of AMRs using whole-cell MALDI-TOF would be a more appropriate option, because whole-cell MALDI-TOF analysis is already part of the bacterial identification process and no extra procedure is required. MALDI-TOF artificial intelligence (AI) aims to extract additional information from the data we already have, rather than replacing the current regular antimicrobial susceptibility testing (AST) methods. It is important to note that this approach cannot fully replace regular AST.

**TABLE 1:** Summary of related works

Reference	Antibiotic(s)	Data (R/S)^*a*^	Performance (ACC/AUC)	Classifier
22	Ciprofloxacin, gentamicin, cefotaxime, piperacillin-tazobactam	71 (blood cultures)		MBT-ASTRA
24	Cefotaxime	19/39 (blood cultures)	0.94	ClinPro Tools
23	Amoxicillin	29/32; 40/63	0.97; 0.95	MBT-ASTRA
	Cefotaxime	30/27; 89/11	0.94; 0.84	

a R, resistant; S, susceptible.

For a quick prediction of AMR, it is necessary to identify E. coli resistance to antibiotics using MALDI-TOF MS. Identifying the relevant mass peaks related to AMRs while also understanding the mechanism of the important protein expression patterns that render E. coli resistant to antibiotics is critical. The primary goal of this study was to find important mass spectra for identifying E. coli resistance to five antibiotics: AMC, CAZ, CIP, CRO, and CXM. As a result, we used MALDI-TOF MS to create antibiotic resistance prediction models for E. coli and examined the common or differential peaks in the case of each antibiotic drug.

## RESULTS

### Analysis of MS spectra.

**FIG 1:**
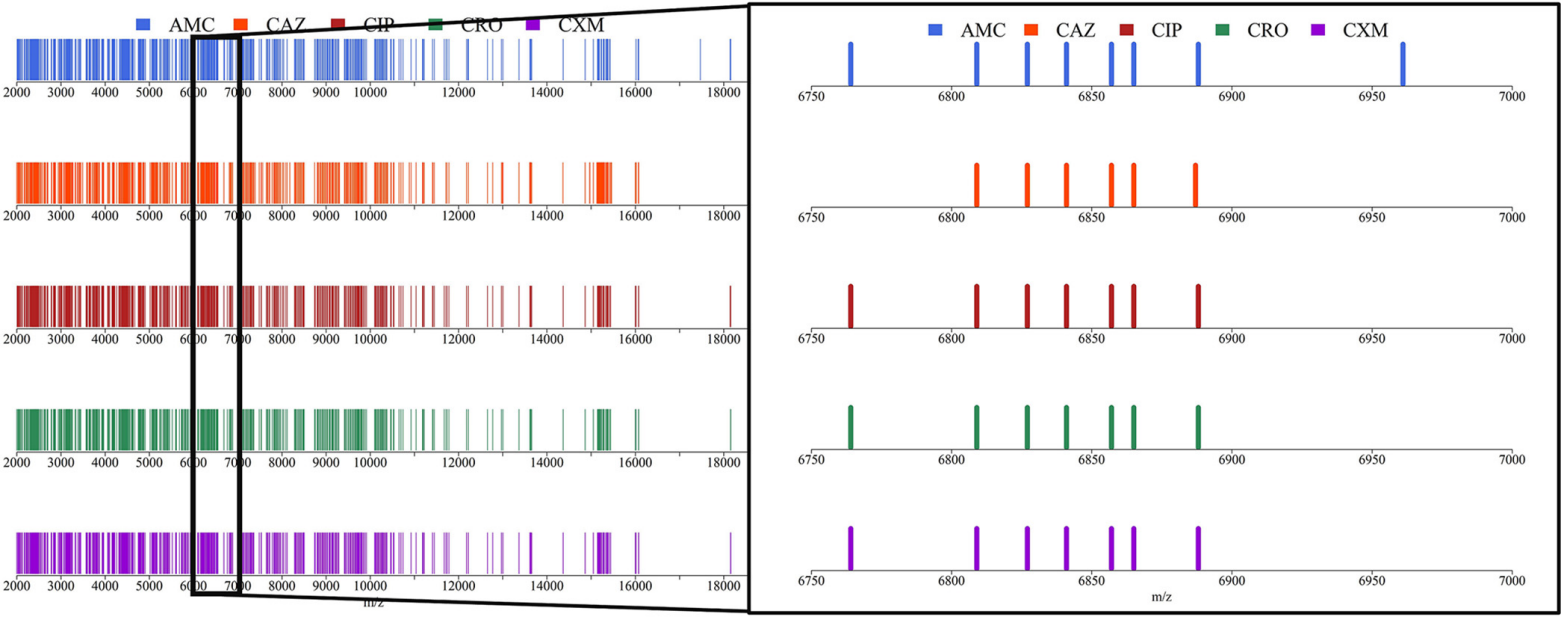
Illustration of the informative peaks (between *m/z* 6750 and 7000) for the samples resistant to five different antibiotics. A colored bar means that the peak was extracted at this *m/z* according to the kernel density estimation. For example, a peak of *m/z* 6764 is absent in a sample resistant to CAZ, and a peak of *m/z* 6961 is only observed for the sample resistant to AMC.

The total peaks for each antibiotic are listed in Table 2. The peaks are the features that we included in the models. Almost all peaks were in the intersection set, except for AMC and CAZ. We then obtained sufficient samples without significant differences between their population distributions. Figure 1 shows the peak positions of the five isolates with different antibiotic resistances. The peak at *m/z* 6764 was not a discriminative peak for detecting CAZ resistance, whereas the peak at *m/z* 6961 was selected as the discriminative peak for AMC, but not other antibiotics.

**TABLE 2:** Summary statistics of peaks for each antibiotic

	No. of peaks		
Antibiotic	Total	Unique presence^*a*^	Unique absence^*b*^
AMC	481	21	11
CAZ	487	32	28
CIP	478	3	3
CRO	476	0	2
CXM	474	1	4
Total^*c*^	431		

a Number of peaks found only for this antibiotic.

b Number of peaks absent only for this antibiotic.

c Number of peaks that all 5 antibiotics have.

**FIG 2:**
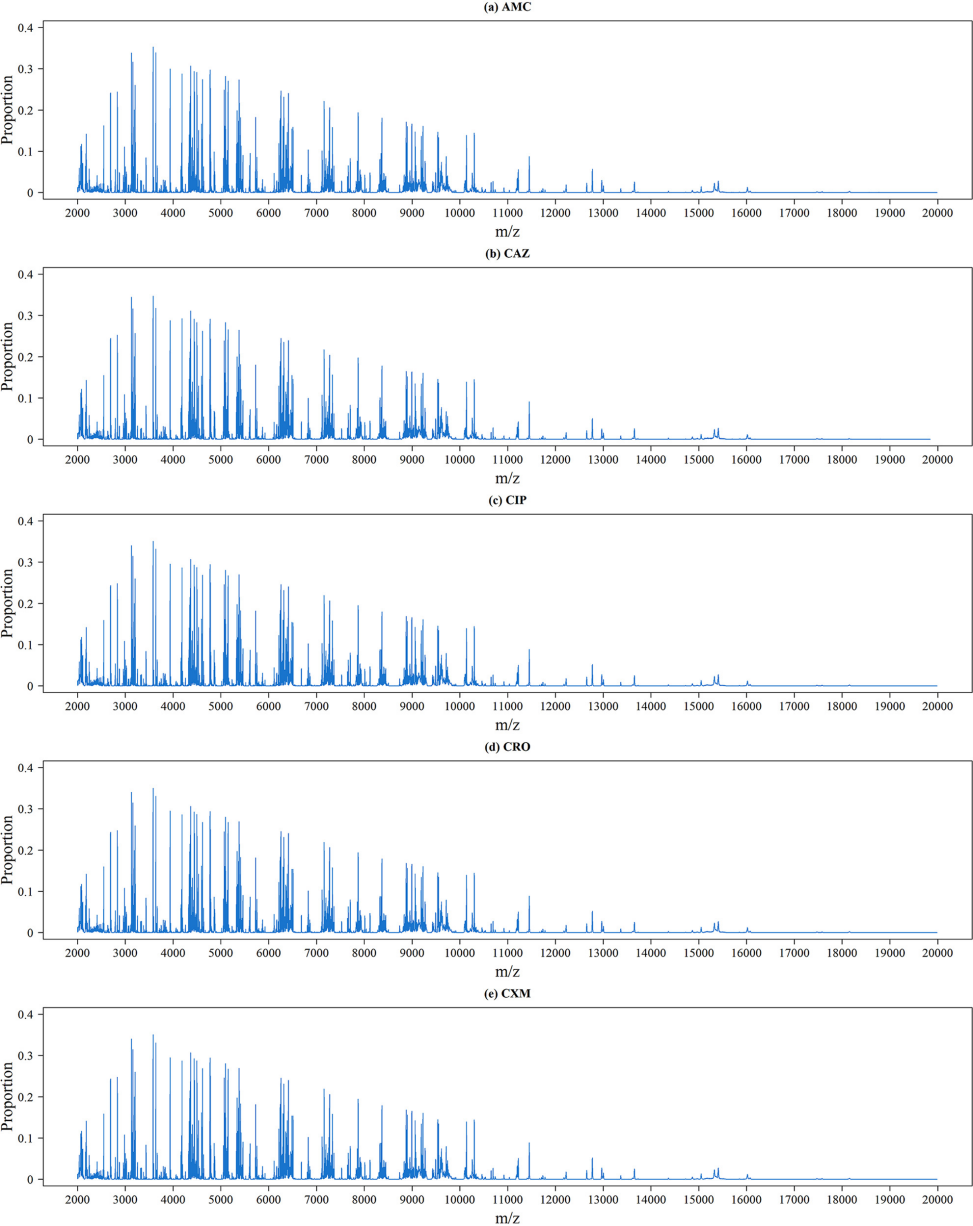
Mass spectrometry of peak distribution of the five antibiotics analyzed in this study.

**FIG 3:**
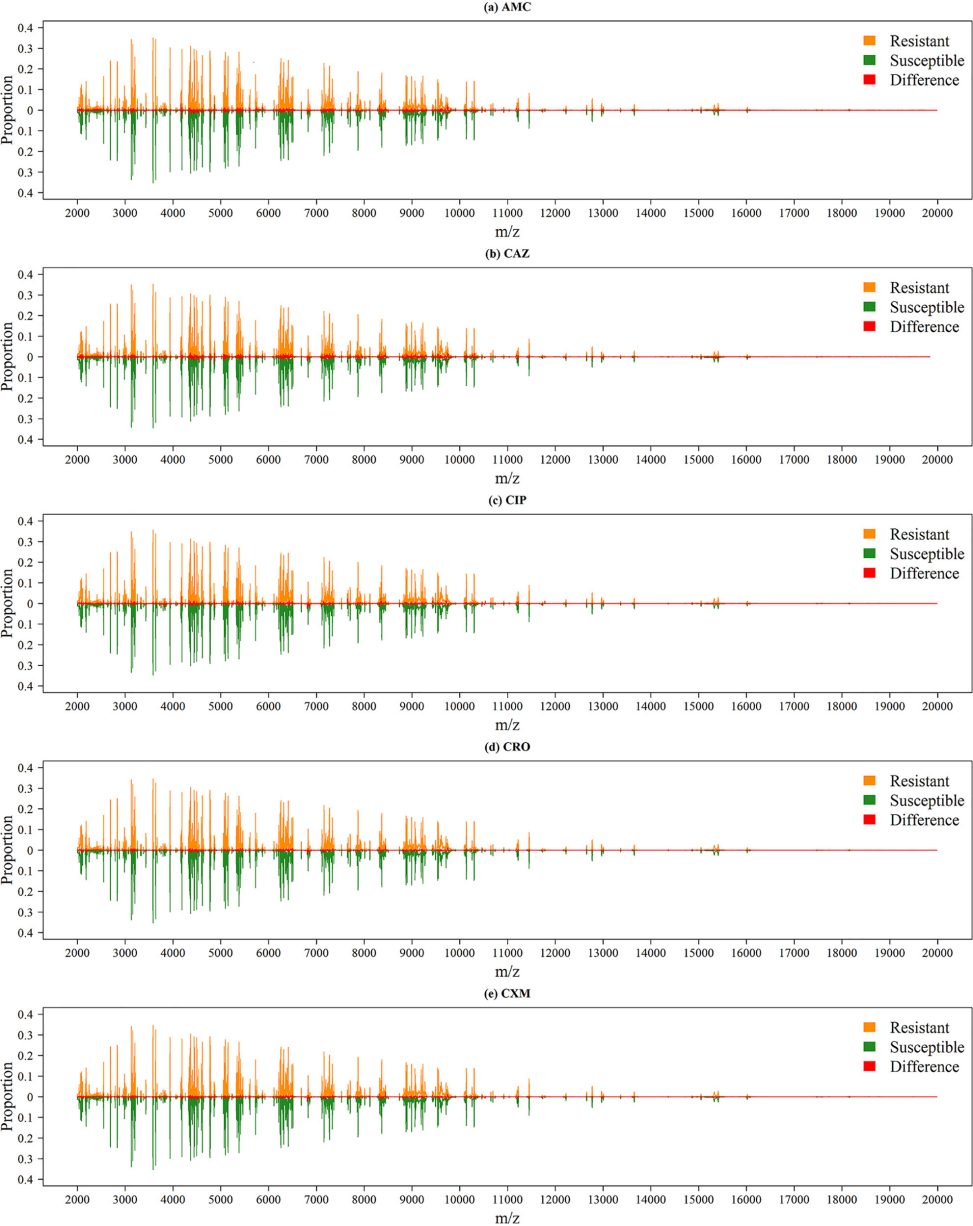
Comparison of spectra of the resistant and susceptible strains for the five antibiotics.

**FIG 4:**
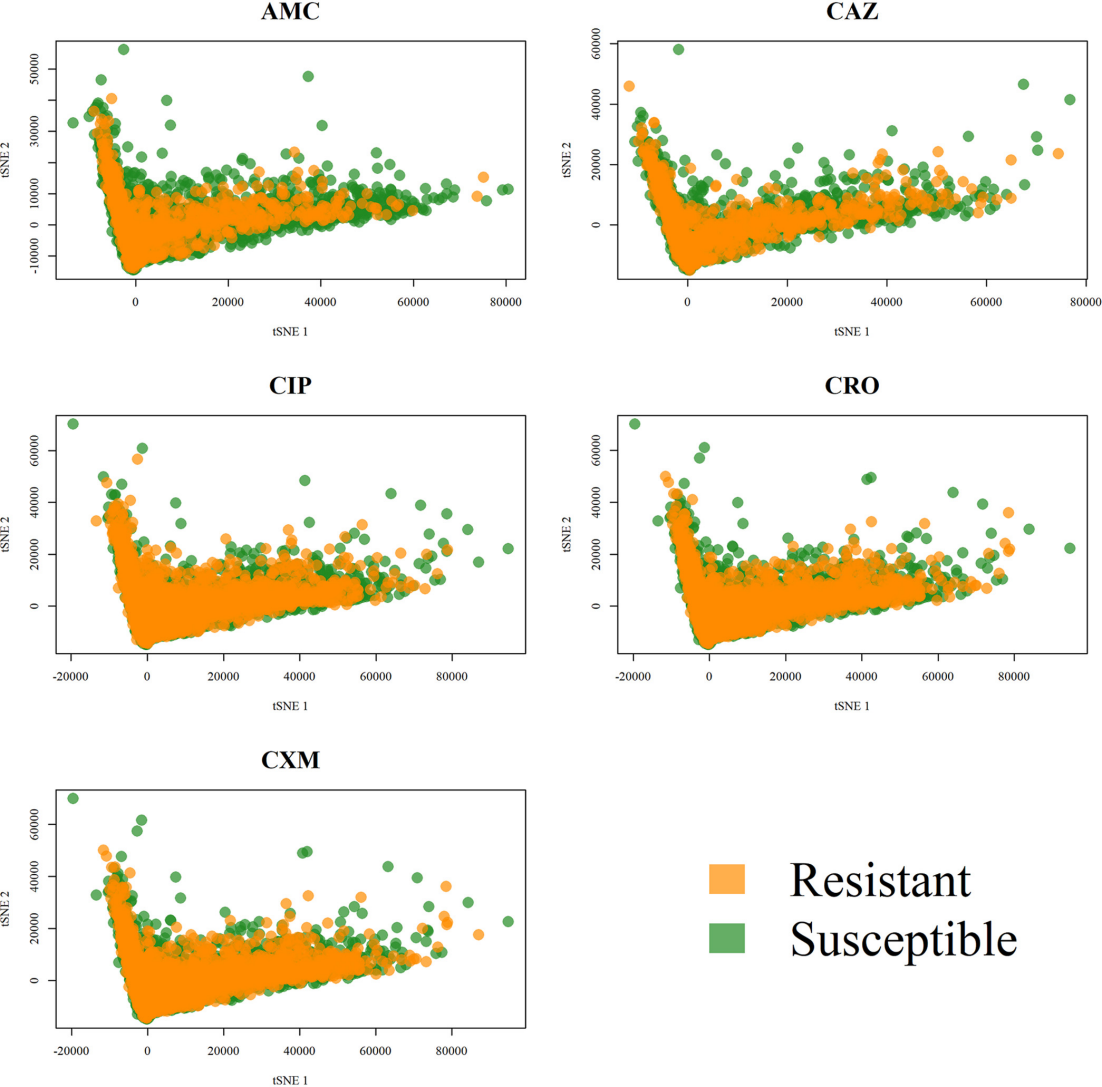
Scatter plots for t-distributed stochastic neighbor embedding with two embedded spaces on five antibiotics.

Figure 2 shows the peak distributions for these five antibiotics; the *x* axis and *y* axis show the *m/z* and the proportion of isolates with this *m/z*, respectively. Because the dimension of the data derived from the MS spectra was high, it was difficult to identify the difference between these five antibiotics. The distributions of the MS spectra for resistant and susceptible isolates are shown in Fig. 3. Specifically, we calculated the proportions of the presence of each *m/z* value from resistant and susceptible isolates. The upper and lower portions of each panel represent the values derived from resistant and susceptible strains. In addition, the difference between them is indicated by a red line. To visualize high-dimensional peaks, we adopted t-distributed stochastic neighbor embedding (tSNE) to further demonstrate the complexity of the data. Figure 4 shows the results of tSNE with two embedded spaces. Because of the complexities in MS data, it is difficult to discriminate between resistant and susceptible isolates directly. In other words, we could not find any apparent areas or peaks that were distinctly representative of resistance or sensitivity. Analysis of other antibiotics showed similar challenges. Therefore, we performed feature extraction to determine more informative features.

### Performance of prediction.

First, we used four machine learning methods for prediction, with the data derived from preprocessing before stage 1. The standard deviations of all the models were not too large. This means that the five subsets of the cross-validation did not differ significantly. Among the four machine learning methods, random forest (RF) and extreme gradient boosting (XGBoost) had higher values of accuracy (ACC) and area under the receiver operating characteristic (ROC) curve (AUC) than the other methods. The details are presented in Table 3.

**TABLE 3:** Performance of four machine learning algorithms on 5-fold cross validation without adopting a two-stage strategy^*a*^

Antibiotic	Metric	Value with algorithm			
		LR	RF	SVM	XGB
AMC	ACC (%)	60.63 ± 1.62	63.24 ± 1.44	58.69 ± 0.79	61.34 ± 0.99
	SEN (%)	60.55 ± 1.59	59.41 ± 1.70	58.64 ± 0.72	61.27 ± 0.95
	SPE (%)	60.65 ± 1.62	64.03 ± 1.40	58.70 ± 0.81	61.35 ± 1.00
	AUC	0.648 ± 0.017	0.652 ± 0.014	0.62 ± 0.011	0.659 ± 0.013

CAZ	ACC (%)	61.08 ± 0.83	61.97 ± 1.71	61.08 ± 1.19	61.22 ± 1.25
	SEN (%)	61.03 ± 0.83	58.26 ± 0.86	61.07 ± 1.11	61.15 ± 1.23
	SPE (%)	61.10 ± 0.83	63.43 ± 1.92	61.09 ± 1.23	61.25 ± 1.26
	AUC	0.657 ± 0.008	0.659 ± 0.016	0.658 ± 0.012	0.664 ± 0.013

CIP	ACC (%)	66.77 ± 1.27	68.55 ± 1.14	65.97 ± 1.47	69.29 ± 1.26
	SEN (%)	66.75 ± 1.28	66.62 ± 1.26	65.95 ± 1.47	69.27 ± 1.26
	SPE (%)	66.78 ± 1.27	69.90 ± 1.39	65.98 ± 1.47	69.31 ± 1.26
	AUC	0.741 ± 0.015	0.764 ± 0.013	0.731 ± 0.018	0.774 ± 0.015

CRO	ACC (%)	62.33 ± 0.79	63.62 ± 0.79	62.42 ± 0.46	63.46 ± 1.17
	SEN (%)	62.32 ± 0.78	61.05 ± 0.97	62.38 ± 0.45	63.45 ± 1.17
	SPE (%)	62.33 ± 0.78	65.04 ± 0.848	62.45 ± 0.47	63.47 ± 1.17
	AUC	0.673 ± 0.008	0.69 ± 0.011	0.67 ± 0.008	0.695 ± 0.014

CXM	ACC (%)	62.61 ± 0.77	63.97 ± 1.16	62.71 ± 0.72	64.20 ± 0.78
	SEN (%)	62.60 ± 0.79	61.33 ± 0.92	62.69 ± 0.71	64.19 ± 0.78
	SPE (%)	62.62 ± 0.76	65.50 ± 1.39	62.72 ± 0.72	64.20 ± 0.793
	AUC	0.678 ± 0.008	0.695 ± 0.013	0.674 ± 0.008	0.701 ± 0.009

a Data are means and standard deviations. AMC, amoxicillin; CAZ, ceftazidime; CIP, ciprofloxacin; CRO, ceftriaxone; CXM, cefuroxime; ACC, accuracy; SEN, sensitivity; SPE, specificity; AUC, area under the receiver operating characteristics curve; LR, logistic regression; RF, random forest; SVM, support vector machine; XGB, extreme gradient boosting.

The performance of the prediction model was poor. Therefore, according to the RF feature importance, we split the data set to make it unique for training. The four models other than the AMC model with informative peaks showed significant improvements in ACC and AUC. The AUC performance of CIP increased from approximately 0.75 to 0.82. The AUC performances of CRO and CXM increased from approximately 0.68 to 0.72. The AUC for CAZ increased from approximately 0.65 to 0.67. However, the performance of AMC did not improve further. In addition, each standard deviation increased slightly owing to the reduction in the number of data sets. The numbers of samples with the presence of informative peaks for each model were 10,188 for AMC, 19,426 for CIP, 5,685 for CAZ, 19,405 for CRO, and 19,150 for CXM. The performance details of the informative peak presence models are listed in Table 4. On the other hand, the performance of models excluding informative peaks decreased. The AUC of the five models was approximately 0.65. The data numbers, excluding informative peaks of each model, were 9,279 for AMC, 16,635 for CIP, 6,578 for CAZ, 16,649 for CRO, and 16,210 for CXM. The detailed performances of the models without informative peaks are presented in Table S4 in the supplemental material.

**TABLE 4:** Performance of informative peak presence models on 5-fold cross validation using a two-stage strategy^*a*^

Antibiotic	Metric	Value with algorithm			
		LR	RF	SVM	XGB
AMC	ACC (%)	60.65 ± 1.47	63.14 ± 1.87	57.82 ± 1.30	60.89 ± 1.95
	SEN (%)	60.43 ± 1.33	59.14 ± 2.09	57.85 ± 1.43	60.85 ± 1.96
	SPE (%)	60.68 ± 1.50	63.91 ± 1.99	57.81 ± 1.28	60.90 ± 1.94
	AUC	0.640 ± 0.023	0.654 ± 0.022	0.609 ± 0.014	0.657 ± 0.016

CAZ	ACC (%)	61.00 ± 1.50	63.85 ± 2.01	61.75 ± 2.77	61.63 ± 2.68
	SEN (%)	60.81 ± 1.49	61.35 ± 1.87	61.57 ± 2.85	61.53 ± 2.63
	SPE (%)	61.06 ± 1.52	64.60 ± 2.01	61.81 ± 2.76	61.66 ± 2.70
	AUC	0.664 ± 0.019	0.673 ± 0.022	0.667 ± 0.024	0.676 ± 0.033

CIP	ACC (%)	72.92 ± 1.40	74.13 ± 1.24	72.25 ± 1.56	74.98 ± 1.58
	SEN (%)	72.88 ± 1.39	72.63 ± 1.59	72.25 ± 1.55	74.94 ± 1.58
	SPE (%)	72.95 ± 1.41	75.34 ± 1.04	72.26 ± 1.56	75.00 ± 1.57
	AUC	0.814 ± 0.015	0.830 ± 0.011	0.803 ± 0.016	0.841 ± 0.014

CRO	ACC (%)	66.28 ± 0.98	67.78 ± 1.13	66.52 ± 0.85	67.30 ± 0.96
	SEN (%)	66.24 ± 0.98	66.10 ± 1.57	66.48 ± 0.84	67.24 ± 0.97
	SPE (%)	66.30 ± 0.98	68.56 ± 0.98	66.54 ± 0.86	67.33 ± 0.96
	AUC	0.720 ± 0.014	0.736 ± 0.015	0.72 ± 0.013	0.74 ± 0.014

CXM	ACC (%)	66.48 ± 1.04	68.01 ± 1.24	66.89 ± 1.01	67.46 ± 1.24
	SEN (%)	66.45 ± 1.04	65.88 ± 1.58	66.76 ± 1.08	67.46 ± 1.23
	SPE (%)	66.50 ± 1.04	69.04 ± 1.17	66.95 ± 0.97	67.46 ± 1.24
	AUC	0.724 ± 0.014	0.738 ± 0.011	0.724 ± 0.012	0.741 ± 0.015

a Data are means and standard deviations. AMC, amoxicillin; CAZ, ceftazidime; CIP, ciprofloxacin; CRO, ceftriaxone; CXM, cefuroxime; ACC, accuracy; SEN, sensitivity; SPE, specificity; AUC, area under the receiver operating characteristics curve; LR, logistic regression; RF, random forest; SVM, support vector machine; XGB, extreme gradient boosting. In the first stage, the informative peak for AMC is *m/z* 4533, and that for the other four antibiotics is *m/z* 9714.

**FIG 5:**
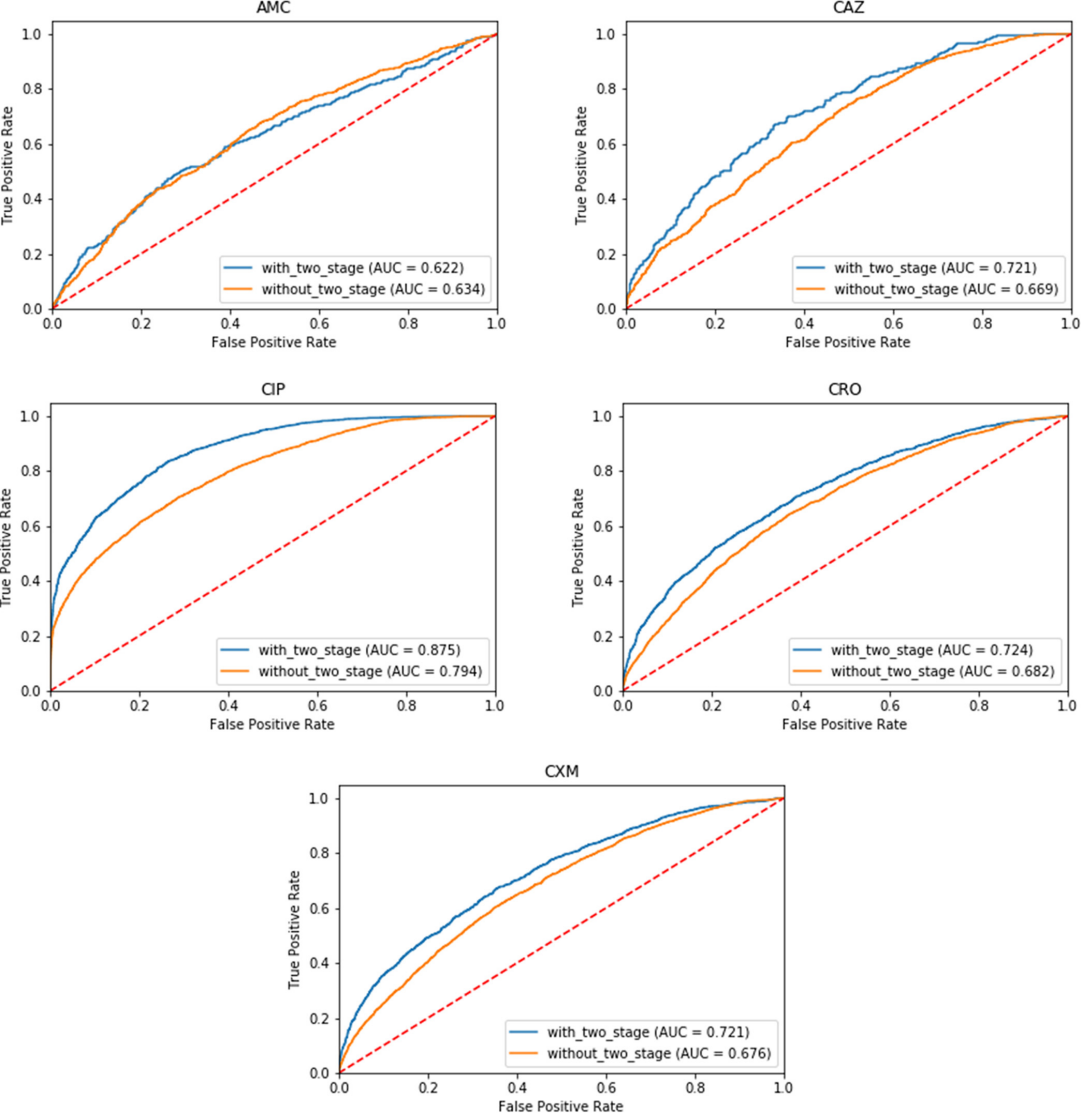
Receiver operating characteristic curves of 5 antibiotics models with (blue line) or without (orange line) two-stage modeling on the independent testing set.

Subsequently, the XGBoost model was tuned. A comparison is presented in Table 5. First, performance did not decline in the independent test on the Kaohsiung data set. Only a slight imbalance between sensitivity (SEN) and specificity (SPE) was noted. According to the experiment results, the AUC of all the models had slightly increased from 0.01 to 0.02 after tuning. However, the imbalance between SEN and SPE was more severe. The numbers of samples with the presence of informative peaks for each model were 3073 for AMC, 4924 for CIP, 1510 for CAZ, 4874 for CRO, and 4853 for CXM on the independent test dataset. Finally, we plotted the ROC curve to compare the improvement after two-stage model training and model tuning in the independent test. For each antibiotic, three ROC curves were plotted for comparison. Three models for each antibiotic were trained using all data, excluding cross-validation (CV), to make the AUC performance slightly different from the average performance of 5-fold CV. The all-data model, without_two_stage models, was trained using the default settings of the XGBoost model. The with_two_stage models, the final models we built, were trained using the tuned hyperparameter setting XGBoost. We could see that, except for AMC, the two-stage model training significantly improved the performance. Moreover, hyperparameter tuning slightly improved AUC performance. The ROC curves for the five antibiotic models are shown in Fig. 5.

**TABLE 5:** Performance of XGBoost models with two-stage modeling on the independent test^*a*^

Antibiotic	Tuning	ACC (%)	SEN (%)	SPE (%)	AUC
AMC	Default	62.23	52.48	63.83	0.61
	Tuned	80.76	22.50	90.27	0.622
CIP	Default	76.40	82.56	70.88	0.865
	Tuned	78.04	79.51	76.72	0.875
CAZ	Default	64.29	61.23	64.87	0.689
	Tuned	77.54	41.73	84.38	0.72
CRO	Default	66.54	61.64	68.48	0.711
	Tuned	71.60	50.87	79.77	0.724
CXM	Default	66.41	59.63	69.24	0.708
	Tuned	69.91	50.98	77.81	0.721

a AMC, amoxicillin; CAZ, ceftazidime; CIP, ciprofloxacin; CRO, ceftriaxone; CXM, cefuroxime; ACC, accuracy; SEN, sensitivity; SPE, specificity; AUC, area under the receiver operating characteristics curve; LR, logistic regression; RF, random forest; SVM, support vector machine; XGB, extreme gradient boosting.

### Informative-peak analysis.

**FIG 6:**
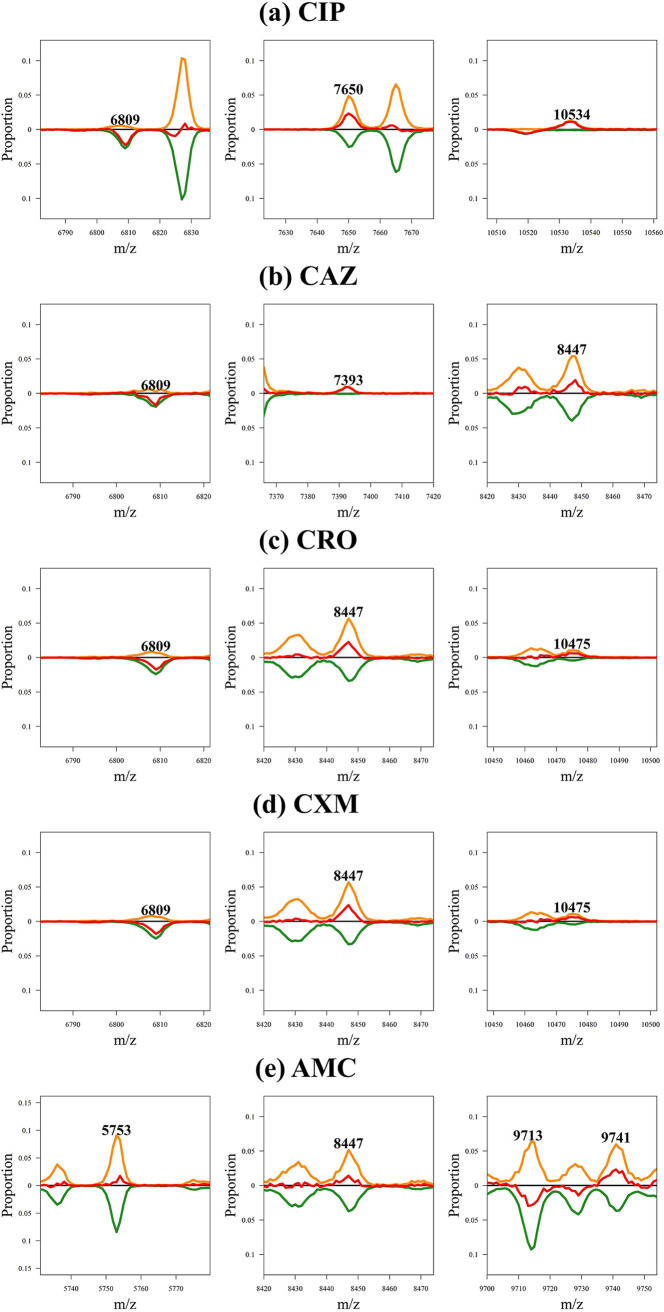
Spectral distribution of the important peaks for (a) CIP, (b) CAZ, (c) CRO, (d) CXM, and (e) AMC based on a random forest classifier.

The CIP model showed the greatest improvement. According to the feature importance of RF, *m/z* 11783, *m/z* 7650, *m/z* 10534, and *m/z* 6809 were the top four prominent peaks. This meant that if the sample had each of these four peaks, it could be identified precisely. However, the proportions of *m/z* 11783, *m/z* 7650, *m/z* 10534, and *m/z* 6809 in all samples were 4.3%, 18%, 4.4%, and 9.9%, respectively, in the CIP data. This value was very low, and most of the samples did not exhibit these peaks. The spectral distributions of the four informative peaks are shown in Fig. 6. Figure 6a shows the discriminative abilities of nearly all samples, with *m/z* 10534 and *m/z* 11783 indicating susceptibility to CIP. Nearly all samples with *m/z* 6809 were resistant to CIP, and most samples with *m/z* 7650 were susceptible to CIP. We also observed that the proportions of the four peaks were low, particularly at *m/z* 10534 and *m/z* 11783. In addition, CAZ, CRO, and CXM exhibited the same two informative peaks at *m/z* 6809 and *m/z* 8447. Other informative peaks, such as *m/z* 7393 for CAZ and *m/z* 10475 for CRO and CXM, had a low proportion. Unfortunately, AMC showed several different peaks for the resistant and sensitive samples; however, no prominent peak was observed. The spectral distributions of the important peaks for CAZ, CRO, CXM, and AMC are shown in Fig. 6b to e, respectively.

**FIG 7:**
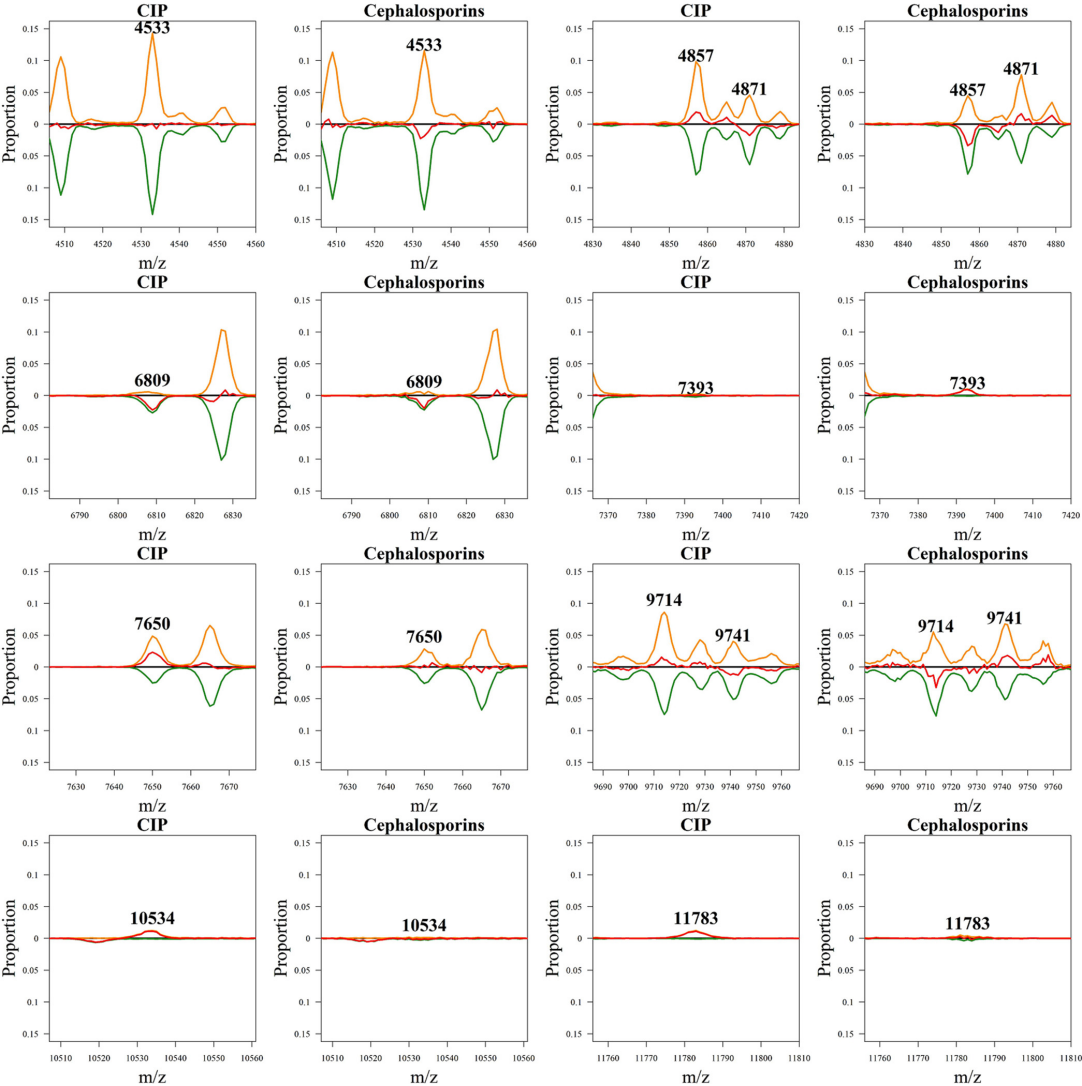
Comparison of the informative peaks between cephalosporins and CIP.

CAZ, CRO, and CXM belong to the same class of antibiotics as cephalosporins. They have similar mechanisms of action and beta-lactam ring structures. Therefore, we used isolates that are resistant or susceptible to CAZ, CRO, and CXM to build a model. We obtained values of 69.37, 65.44, 70.42, and 0.756 for ACC, SEN, SPE, and AUC, respectively. We also found informative peaks related to cephalosporin resistance. Most importantly, Fig. 7 shows that the informative peaks for cephalosporins and CIP were reversed. The CIP data showed no difference between the resistant and susceptible peaks at *m/z* 4533 and 7393, which are cephalosporin-informative peaks. In addition, *m/z* 4857, 4871, 9714, and 9741 appeared distinctly in the cephalosporins and CIP. In contrast, cephalosporins showed no difference between resistant and sensitive peaks at *m/z* 7650, 10534, and 11783, which were CIP-informative peaks. All antibiotics had *m/z* 6809 peaks, regardless of their mechanism.

We also evaluated the independence between antibiotic resistance and the appearance of important peaks using odds ratios (ORs). We calculated the ratio of resistance to sensitivity in the presence and absence of such informative peaks. We then divided it to obtain the OR. A higher value indicates a higher peak, representing resistance or sensitivity. The odds ratio results of the informative peaks and CIP resistance are listed in Table 6. The informative peaks were significantly related to CIP (Table 6). The ORs of *m/z* 6809, *m/z* 7650, *m/z* 10534, and *m/z* 11783 were 3.4, 2.24, 6.98, and 9.77, respectively. ORs greater than 1 indicate that these peaks are related to CIP resistance, and *m/z* 6809 is related to CIP susceptibility. The odds ratios of informative peaks in the case of other antibiotics were also significant. Considering CIP as an example, after understanding the importance of these peaks, we further considered the relationship between the *m/z* 9714 peak and these four peaks. Most of the four informative peaks appeared at *m/z* 9714 at the same time. Ninety percent of the isolates with the *m/z* 7650 peak had *m/z* 9714, 87% of the samples with the *m/z* 10534 peak had *m/z* 9714, 83% of the samples with the *m/z* 11783 peak had *m/z* 9714, and 74% of the samples with the *m/z* 6809 peak had *m/z* 9714. Although the subset with peak at *m/z* 9714 contained most of the four informative peaks, the proportion of samples with informative peaks was still low. The proportion of the four informative peaks in the subset with *m/z* 9714 was 13.5% for *m/z* 6809, 29.8% for *m/z* 7650, 7.1% for *m/z* 10534, and 6.6% for *m/z* 11783. We also evaluated the independence between the appearance of the four informative peaks and *m/z* 9714 by using the odds ratio. The odds ratio results for the informative peaks are listed in Table 7. We found that the odds ratios for four informative peaks and the *m/z* 9714 peaks were greater than 1. Therefore, we inferred that the subset with the *m/z* 9714 peaks had more informative peaks. This makes a subset with informative peaks discriminative. The odds ratio results of the informative peaks for other antibiotics, CAZ, CRO, and CXM, also showed a similar proportion and significance of informative peaks in the subset, except for *m/z* 7393 in the CAZ subset.

**TABLE 6:** Odds ratio results in informative peaks and antibiotic resistance^*a*^

Antibiotic	Sample characteristic	No. of samples		OR
		R	S	
CIP	Presence of 6809	667	2,950	3.4
	Absence of 6809	14,262	18,533	
	Presence of 7650	3,774	2,813	2.24
	Absence of 7650	11,155	18,675	
	Presence of 10534	1,310	292	6.98
	Absence of 10534	13,619	21,196	
	Presence of 11783	1,360	218	9.77
	Absence of 11783	13,569	21,270	
CAZ	Presence of 6809	157	855	2.32
	Absence of 6809	3,398	7,946	
	Presence of 7393	171	45	9.83
	Absence of 7393	3,384	8,756	
	Presence of 8447	1,236	2,346	1.46
	Absence of 8447	2,319	6,455	
CRO	Presence of 6809	700	2,923	2.5
	Absence of 6809	12,308	20,483	
	Presence of 10475	959	711	2.54
	Absence of 10475	12,049	22,695	
	Presence of 8447	4,524	5,736	1.64
	Absence of 8447	8,484	17,670	
CXM	Presence of 6809	699	2,875	2.56
	Absence of 6809	12,358	19,778	
	Presence of 10475	970	675	2.61
	Absence of 10475	12,087	21,978	
	Presence of 8447	4,506	5,468	1.65
	Absence of 8447	8,551	17,185	

a R, resistance; S, susceptibility. The formula for the OR is (A/B)/(C/D), where A is the R of presence of peak, B is the S of presence of peak, C is the R of absence of peak and D is the S of absence of peak. Note that the formula for 6809 is (B/A)/(D/C).

**TABLE 7:** Informative peaks detail of CIP, CAZ, CRO, and CXM in informative peak subset^*a*^

Antibiotic	Sample characteristic	No. of samples with:		OR
		Presence of 9714	Absence of 9714	
CIP	Presence of 6809	2,680	942	2.61
	Absence of 6809	17,102	15,693	
	Presence of 7650	5,913	674	10.09
	Absence of 7650	13,869	15,961	
	Presence of 10534	1,405	197	6.37
	Absence of 10534	18,377	16,438	
	Presence of 11783	1,319	259	4.51
	Absence of 11783	18,463	16,376	

CAZ	Presence of 6809	705	307	2.83
	Absence of 6809	5,073	6,271	
	Presence of 7393	59	157	0.42
	Absence of 7393	5,719	6,421	
	Presence of 8447	2,003	1,579	1.67
	Absence of 8447	3,775	4,999	

CRO	Presence of 6809	2,684	939	2.62
	Absence of 6809	17,081	15,710	
	Presence of 10475	1,416	254	4.98
	Absence of 10475	18,349	16,395	
	Presence of 8447	6,323	3,937	1.51
	Absence of 8447	13,442	12,712	

CXM	Presence of 6809	2,653	921	2.61
	Absence of 6809	16,847	15,289	
	Presence of 10475	1,396	249	4.94
	Absence of 10475	18,104	15,961	
	Presence of 8447	6,217	3,757	1.55
	Absence of 8447	13,283	12,453	

a The formula for the OR is (A/B)/(C/D), where A is the value for presence of 9714 and presence of the indicated peak, B is the value for absence of 9714 and presence of the indicated peak, C is the value for presence of 9714 and absence of the indicated peak, and D is the value for absence of 9714 and absence of the indicated peak.

## DISCUSSION

With the growing concern about AMR and superbugs, we need to predict or identify drug-resistant microorganisms quickly and identify the important MS peaks that characterize them. Previous studies identified resistant E. coli isolates by comparing the area under the plasma drug concentration-time curve to calculate the growth rate between samples incubated with or without antibiotics (22–24). The peak around *m/z* 6800 also indicates that it does not react with antibiotic incubation (23). Compared to antibiotic hydrolysis testing by MALDI-TOF, whole-cell MALDI-TOF is more promising because no additional antibiotic material or testing time is required for a preliminary AST. In addition, the informative peaks that we found were highly related to resistance, which could provide indications for further research.

We believe that several factors (such as accuracy, turnaround time, and cost) can influence clinical microbiological testing techniques. However, many microbiological tests, including regular AST, are far from perfect and can be time-consuming. As the most important factor in life-threatening infections is time, we propose a MALDI-TOF-based approach that can improve antibiotic administration at an earlier time point. MALDI-TOF AI requires only whole-cell MALDI-TOF MS spectra for AMR prediction and does not require any additional wet lab work or time. AMR predictions can be generated within seconds after the bacterial species have been identified. Using this approach, the accuracy of empirical antibiotic use can be improved from approximately 50% (when the resistant-to-susceptible ratio is approximately 1:1) to over 80%. Guidelines often require accuracy rates of 90% or higher. However, an improvement of approximately 30% in antibiotic use without added costs can provide more useful information for effectively managing patients earlier and is worthy of further investigation.

In this study, we focused on commonly used treatments, including ciprofloxacin, cefuroxime, ceftriaxone, ceftazidime, and amoxicillin. Additionally, the ratio of resistant to susceptible strains for these antibiotics was approximately 1:1, which would result in an accuracy rate of approximately 50% if empirical antibiotic use was based on this ratio. This accuracy rate is far from ideal, and it is an unmet need that we aimed to address in this study. Although a predictive performance of 80% accuracy is still not perfect, it would represent a significant improvement over the current 50% accuracy rate. In contrast, the resistance frequency for certain last-resort drugs, such as carbapenems, is still relatively low. In Taiwan, carbapenem-resistant E. coli accounts for only a small percentage of cases. Using carbapenem as an empirical antibiotic treatment based on this information would result in a high level of accuracy, and the current MALDI-TOF AI technology may not add much value in this context. To our knowledge, the highest accuracy achieved in a large cohort for discriminating MRSA and methicillin-susceptible S. aureus (MSSA) using MALDI-TOF AI technology was around 95% in previous studies from our team (15). However, for E. coli, no antibiotic, including last-resort drugs, has achieved levels of performance as high as the predicted performance. This suboptimal performance suggests that the complexity of drug resistance in E. coli is still not well understood using MALDI-TOF MS and AI technologies. In addition, local epidemiological data on resistance rates, such as those for carbapenems, may be more accurate and helpful in this context than using MALDI-TOF AI.

The use of MALDI-TOF MS spectra to predict antibiotic resistance has the potential to provide rapid and accurate drug resistance information several days before conventional testing methods. This early information on drug resistance could significantly improve patient survival rates. To generate reliable predictions, it is necessary to standardize the various steps in the process, including sample preparation, MALDI-TOF analysis, MS data preprocessing (25), and model training and validation. As sample preparation would be the most original and crucial step, we are still investigating how to standardize the preparation step. Working in an area with highly fluctuating humidity, we noted that humidity affected sample preparation and MALDI-TOF analysis. To address this issue and improve the standardization of sample preparation, we plan to use the MBT FASTTM Shuttle device to perform the preparation step before MS analysis in further research.

As an application study, our study attempted to predict antimicrobial resistance based on the existing MALDI-TOF MS spectra that had already been generated for bacterial species identification. In the regular workflow of the clinical microbiology laboratory, we did not include an internal calibrator for the generation of these spectra. As noted, the MALDI-TOF MS technique that is commonly used in clinical microbiology laboratories may not be sufficiently precise. We encountered difficulties in identifying peaks using either the LIFT technique or liquid chromatography-tandem MS (LC-MS/MS) and have been unsuccessful in achieving high-confidence peptide identification using *de novo* assembly based on secondary MS information. One potential reason for this could be the presence of a larger number of peptide species at specific locations in the MS spectra than expected. MALDI-TOF MS with higher resolution might help address this issue. Another possibility is the presence of posttranslational modifications of the peptides, which can increase the complexity of the assembly from MS/MS fragments. While we continue to work on these challenges, we would like to share a list of peaks in the hope that the proteomics community can help in these efforts.

In previous related studies, the most informative peaks were from ribosomal proteins or abundant housekeeping proteins. Unintuitively, peptides or proteins that have been considered important for antibiotic resistance delivery have rarely been reported as informative peaks. It is an interesting phenomenon that we do not understand based on current proteomic technologies for sequencing peptides. One possible explanation is that only abundant peptides/proteins are associated with specific locally resistant strains. The pattern of peptides/proteins is an epidemiological snapshot. MALDI-TOF MS is not sufficiently sensitive to detect resistance-related peptides in this setting. Another possible explanation is that the whole pattern of ribosomal and housekeeping proteins would also contribute to antibiotic resistance. To test this hypothesis, we first needed improved peptide sequencing technology to identify each informative peptide. However, this is a labor-intensive and time-consuming task. Current chromatography-MS/MS technology cannot be used for rapid and easy peptide identification. Many factors, including posttranslational modifications, prevent us from knowing the sequences and identities of these peptides. Pore-based direct peptide sequencing technology (e.g., Oxford Nanopore Technology), which is booming, can be used to solve this problem.

In our study, we transformed the MS data obtained from Chang Gung Memorial Hospital (CGMH) to structural data by smoothing, baseline subtraction, and peak selection using flexAnalysis. We then cleansed the data, fitted the distribution of the MS data using Gaussian kernel density, detected the peaks, and aligned the peaks. After feature extraction, we identified the informative peaks and split the data set using the informative peaks in the first stage of model training. Finally, we built such models for the informative peak subset and tuned them to obtain the best AUC values of 0.622, 0.875, 0.72, 0.724, and 0.721 for AMC, CIP, CAZ, CRO, and CXM, respectively, for the XGBoost model. Moreover, we further analyzed the informative peaks of the results based on feature importance. The informative peak at *m/z* 9714 appeared with some important peaks at *m/z* 6809, *m/z* 7650, *m/z* 10534, and *m/z* 11783 for CIP, and *m/z* 6809, *m/z* 10475, and *m/z* 8447 for CAZ, CRO, and CXM. We statistically analyzed the informative peak subset properties and found a difference between the subsets presenting informative peaks on SPC and age. We plan to perform feature extraction or model training using deep learning. The use of deep learning to capture more information from raw MS data, rather than using flexAnalysis or Gaussian kernel density, prevents the loss of continuous data. In addition, more clustering methods were used to split the data into a more specific subset to make each data set unique. Finally, the proper implementation of antibiotics other than CIP is also an important area for future research.

## MATERIALS AND METHODS

### Clinical sample and MALDI-TOF MS data.

All clinical samples were provided by two referral medical centers, namely, the CGMH Linkou and Kaohsiung branches. Samples were collected consecutively from 2013 to 2018 from Linkou and Kaohsiung branches from 2015 to 2017. Five types of specimens were included: blood, urine, respiratory tract, sterile cavity fluid, and wound. For all specimens except blood, we inoculated the specimens on various solid-agar plates and cultured them overnight. Single colonies were picked for MALDI-TOF analysis. Blood samples were obtained from patients in whom bacteremia was suspected and cultured in Trypticase soy broth. Positive blood culture samples were subcultured onto blood agar plates and subjected to a rapid spin-down procedure for MALDI-TOF analysis and AST prediction. The process involved transferring 5 mL of blood from the positive blood culture bottle to a serum separator tube and centrifuging at 3,500 rpm for 10 min. The supernatant was removed, and the pellet was resuspended in 1 mL of 0.9% saline, followed by centrifugation at 10,000 rpm for 5 min. The pellet was mixed with 1 mL red blood cell (RBC) lysis buffer for 3 min and then centrifuged again at 10,000 rpm for 5 min. The precipitate was dried for 3 min before being used for MALDI-TOF analysis. The entire procedure took less than an hour to complete once a positive blood culture was identified.

MALDI-TOF MS was performed to identify the bacterial species, and MS data were analyzed for antibiotic resistance using our model. MALDI-TOF MS measurements were performed according to the manufacturer’s instructions on a Microflex LT mass spectrometer (Bruker Daltonik GmbH, Bremen, Germany) equipped with a 60-Hz nitrogen laser. The parameter settings were set as follows: linear positive mode, accelerating voltage of +20 kV, and 240 laser shots to hit each sample for measurement. Escherichia coli was identified using Biotyper 3.1 (Bruker Daltonik GmbH, Bremen, Germany), and the MALDI-TOF MS spectra were processed using flexAnalysis 3.3 (Bruker Daltonik GmbH, Bremen, Germany). The spectra were smoothed using the Savitzky-Golay algorithm, and the spectrum baseline was subtracted using the top-hat method. The parameters for the peak selection are as follows: the signal-to-noise threshold (SN ratio) was set to 2, the relative intensity threshold was set to 0%, the minimum-intensity threshold was 0, the maximal number of peaks was 200, the peak width was 6 *m/z*, the height was 80%, and the spectral mass ranged from 2,000 to 20,000 *m/z*. The green line graph in Fig. S1 represents one of our E. coli sample MS spectral raw spectra, and the red point in the lower graph represents the chosen peaks after smoothing, baseline subtraction, and peak selection using flexAnalysis. Clinical microbiology laboratories accredited by the College of American Pathologists (CAP) conducted clinical tests following CLSI guidelines. Several quality check-up stages were carried out for MALDI-TOF MS studies following the manufacturer's instructions (Bruker, Bremen, Germany). The Bruker MALDI-TOF mass spectrometer was first calibrated using bacterial test standard (BTS); E. coli BTS (Bruker) was used for calibration. Second, for bacterial identification, Staphylococcus aureus ATCC 25923 was used as an external control to assess whether the analyzer accurately identified those species. A log score of 2.0, generated by Biotyper, was used as the cutoff for the quality check.

Antibiotic susceptibility was evaluated using both microdilution (for blood and sterile cavity fluid specimens) and paper disc methods (for other specimens). The MIC of antibiotics was determined using the M50 and microdilution methods. Antibiotic susceptibility was then interpreted according to the Clinical and Laboratory Standards Institute M100 guidelines. All of the isolates were subjected to CIP, CRO, and CXM ASTs according to the procedures followed by the two medical centers. In contrast, AMC AST was carried out only for isolates from urine specimens, and CAZ AST was tested for isolates from the other four specimen types except for urine. The AST results were the ground truth for training the model. All clinical microbiology tests were conducted following the guidelines of the Clinical and Laboratory Standards Institute, and the clinical microbiology laboratory has been accredited by the CAP since 2003.

### Data analysis and cleansing.

We set the MALDI-TOF MS spectra obtained from the Linkou branch of CGMH as the training set (*n* = 38,595) and the spectra obtained from the Kaohsiung branch as the testing set (*n* = 9,628). We analyzed the training set for feature extraction and provided better input for machine learning. First, we discarded the results labeled as intermediate after AST for each antibiotic and calculated the susceptible proportion, as shown in Table S1. The proportions of each antibiotic were not significantly different between Linkou and Kaohsiung (Table S1). Second, we counted the peaks for each sample derived from flexAnalysis and plotted the distribution using a bar plot. Taking CIP as an example, we found no significant differences between CIP-susceptible and CIP-resistant isolates; however, several isolates showed only a few peaks. We excluded isolates in which the number of peaks was lower than two standard deviations (i.e., 58 peaks) (*n* = 677). These isolates were considered noise. The statistical data for the peaks of each isolate are shown in Table S2. The distribution graph is shown in Fig. S2. Table 8 shows the amount of data after cleansing and the proportions of genders and specimen types in the Linkou branch.

**TABLE 8:** Proportions of gender and specimen type after data cleansing in the Linkou branch

Antibiotic	No. (%) of specimens (*n* = 37,918)							
	Gender			Type^*a*^				
	Total	Female	Male	U	B	W	F	R
AMC	19,730	15,860 (80.38)	3,869 (19.6)	19,718 (99.93)	0	6	1	5
CIP	36,417	24,658 (67.71)	11,750 (32.26)	23,063 (63.33)	5,172 (14.2)	4,097 (11.25)	2,009 (5.51)	1,858 (5.1)
CAZ	12,356	5,844 (47.29)	6,505 (52.64)	22 (0.17)	4,875 (39.45)	3,730 (30.18)	1,857 (15.02)	1,651 (13.36)
CRO	36,414	24,675 (67.76)	11,730 (32.21)	23,044 (63.28)	5,199 (14.27)	4,075 (11.19)	2,010 (5.51)	1,847 (5.07)
CXM	35,710	24,198 (67.76)	11,503 (32.21)	22,673 (63.49)	5,057 (14.16)	3,982 (11.15)	1,954 (5.47)	1,811 (5.07)

a U, urine; B, blood; W, wound; F, sterile cavity fluid; R, respiratory tract.

### Feature extraction.

First, owing to the small difference between sample proteins or peptides, such as atoms, the same peptide, which should have the same *m/z* values, would not have the same *m/z* values. This is referred to as peak shifting (Fig. S3). For Fig. S3, we randomly chose four spectra, two resistant and two susceptible, and showed part of the spectra. The *y* axis represents the intensity. The dots represent the peaks selected by flexAnalysis. All four samples had a peak around *m/z* 2065 for one of the peptides but shifted by approximately 5 *m/z* (14). Moreover, the shifting range is not solidly defined; therefore, we decided to use the Gaussian density to fit the distribution and solve the shifting problem. Second, E. coli populations are highly unstable and genetically diverse. Therefore, we split the data to make each group unique for training purposes. We chose the random forest feature importance as the standard to choose the informative peaks to split the subset.

To address the shift problem, we chose a kernel density estimation to fit the curve and chose a Gaussian distribution for the kernel function. First, we plotted all the spectra together as a histogram and considered each part of the curve as a single protein pattern. We regarded the distribution of each peak as independent and normal. Therefore, we used a kernel density estimation with a Gaussian function. Kernel density estimation calculates the probability density for all data, splitting a histogram and the range according to the kernel function. The kernel density estimation formula is as follows:

$$f(x)=\frac{1}{\mathrm{nh}}\sum_{i=1}^{n}K\left(\frac{x\;-\;x_{i}}{h}\right)$$

where *h* is the bandwidth at which the histogram should be split, *K* is the kernel function, *n* is the number of m/z values, *x* represents the random variable of the peaks, and *x_i_* is the m/z value. We chose the Gaussian function, whose formula is as follows:

$$K(u)=\frac{1}{\sqrt{2\pi }}\exp\left\{-\frac{1}{2}u^{2}\right\}$$

where *K* is the kernel function and *u* is a variable for the kernel density function. We then decided on the bandwidth, which is a hyperparameter. The small bandwidth causes the curve to become undersmoothed. In contrast, a large bandwidth causes oversmoothing. We experimented with the optimal bandwidth to fit the data distribution. Bandwidths of 2 and 3 caused oversmoothing; therefore, we chose 1 as the bandwidth. We used the CIP data as an example, and the experimental comparison is shown in Fig. S4. We utilized the scikit-learn kernel density package for kernel density estimation (26).

After fitting the data distribution, the peaks and their ranges were located. We used the argrelextrema function in the scipy to calculate the relative extrema of the data, in other words, to determine the local maximum and local minimum (27). We then set the local maximum threshold to filter the noise. The remaining local maxima were regarded as peaks, and two local minima were beside the ranges of the peaks (Fig. S5). The red points are the local maxima, which we regard as the peaks. Two points on the left side were deleted because of the threshold. The two colors of the curve discriminate the range of the peaks.

### Two-stage model training.

Building a prediction model to cover all E. coli isolates would be challenging, given the high diversity of the E. coli population. Hence, in stage 1 of the model training, we used the RandomForestClassifier function, provided by the scikit-learn package to build the model (26). We then separated the samples according to the RandomForestClassifier attribute feature_importances, which indicates the importance of features in the classifier task. We chose the peak that ranked high in feature importance and appeared to be sufficiently high in the data set. The appearance proportions and order of feature importance are shown in Fig. S6. In summary, there were very few high-importance peaks. Importance peak scores were not prominent, or the proportion was too low; for example, the proportion of the most important peak *at m/z* 7393 in CAZ was only 1.75%. In this case, we experimented with the top 10 peaks with a higher proportion (>25%), then chose the best peak for each antibiotic based on the performance of RF, and regarded it as an informative peak. We split the data into two groups based on the informative peak presentation. Then, we excluded the subset whose informative peak was absent and trained all the models with subsets whose peak was present in the 5-fold CV. Finally, we chose *m/z* 4533 for the classification of AMC AST and *m/z* 9714 for the other four antibiotics’ AST because of the higher AUC in the validation performance. The 5-fold CV random forest average results for all data sets and data sets with informative peak presence are shown in Table S3. In stage 2, we used subset data with informative peak presence training for four machine learning models: logistic regression, support vector machine (SVM), random forest, and XGBoost. After choosing the best machine learning model based on the AUC performance in a 5-fold CV with the default parameter setting, we further tuned the hyperparameter in the 5-fold CV and trained the final model with the best tuned hyperparameter and all subset data. Most studies investigating MS spectra used decision trees (DT) (14), RF (28, 29), SVM (14, 28, 30–33), neural network (NN) (32, 34), and k-nearest neighbor (KNN) (14, 28). In our study, after clustering, we tested four machine learning methods (LR, SVM, RF, and XGBoost) to classify the resistance of CIP in a 5-fold CV with default parameter settings. Then, we chose the highest-performance model, XGBoost, and tuned the hyperparameters to the best performance in a 5-fold CV. Detailed descriptions of these methods are provided in the supplemental material (machine learning models).

### Evaluation metrics.

In this study, all training performance and hyperparameter tuning were performed using 5-fold cross-validation on the training data from the Linkou branch, except that the final model was trained using all the training data. To be more specific about 5-fold cross-validation, we randomly divided the training set into five subsets, and all subsets had equal amounts of data. In each round of cross-validation, different subsets were selected as the validation set, and the remaining four subsets were combined for the training model. After repeating the experiment five times, we evaluated the model using the following evaluation metrics and averaged it to obtain the final performance.

Four metrics were used to evaluate our model: ACC, SEN, SPE, and AUC. Accuracy is the most common and easy-to-understand evaluation index. This directly reveals the proportion of correct predictions. Accuracy is defined as (TP + TN)/(TP + TN + FP + FN), where TP is true positive, i.e., the number of correctly predicted positive samples; TN is true negative, i.e., the number of correctly predicted negative samples; FP is false positive, i.e., the number of negative samples that were incorrectly predicted; and FN is false negative, i.e., the number of positive samples that were incorrectly predicted.

In addition to accuracy, we were also concerned with the correct proportion of real positive or real negative cases in the data to avoid the problem of unbalanced data. SEN evaluates the performance of classifiers on positive data, and SPE evaluates the performance of the classifier on negative data. Sensitivity and specificity are defined as TP/(TP + FN) and TN/(FP + TN), respectively.

The AUC is the area under the receiver operating characteristic curve. The ROC curve is a graph that shows the performance of the classification model at any threshold. The ROC curve draws two parameters: the true-positive rate (TPR), which is the same as the sensitivity, and the false-positive rate (FPR), which is 1 − specificity. The ROC was plotted using FPR on the *x* axis and TPR on the *y* axis for every threshold. This shows the trade-off between TPR and FPR to infer the model performance on the positive and negative data simultaneously. Therefore, in our study, we focused mainly on AUC performance. For the threshold, we took the minimum TPR – (1 – FPR) threshold, which balanced the SEN and SPE.

### Data availability.

The training and testing data sets have been uploaded onto the GitHub open source. Data and scripts can be accessed freely at https://github.com/chungcr/ABR_E.coli_identification/tree/main/data.
