# Supplementary material for: Data-Driven Two-Stage Framework for Identification and Characterization of Different Antibiotic-Resistant Escherichia coli Isolates Based on Mass Spectrometry Data
Source: Microbiol Spectr. 2023 Apr 12;11(3):e03479-22. doi: 10.1128/spectrum.03479-22 (PMC10269626; doi:10.1128/spectrum.03479-22)
Supplement: Supplemental file 1 — Supplemental material. Download spectrum.03479-22-s0001.pdf, PDF file, 0.7 MB [file spectrum.03479-22-s0001.pdf]

Data-driven two-stage framework for identification and characterization of different antibiotic-resistant *Escherichia coli* based on mass spectrometry data

Chia-Ru Chung[1,†], Hsin-Yao Wang[2,3,†], Chun-Han Yao[1], Li-Ching Wu[4], Jang-Jih Lu[2,5,6,*], Jorng-Tzong Horng[1,7,*],and Tzong-Yi Lee[8,*]

[1]Department of Computer Science and Information Engineering, National Central University, Taoyuan, Taiwan

[2]Department of Laboratory Medicine, Chang Gung Memorial Hospital at Linkou, Taoyuan, Taiwan

[3]Ph.D. Program in Biomedical Engineering, Chang Gung University, Taoyuan, Taiwan

[4]Department of Biomedical Sciences and Engineering, National Central University, Taoyuan, Taiwan

[5]College of Medicine, Chang Gung University, Taoyuan, Taiwan

[6]Department of Medical Biotechnology and Laboratory Science, Chang Gung University, Taoyuan, Taiwan

[7]Department of Bioinformatics and Medical Engineering, Asia University, Taichung, Taiwan

[8]Institute of Bioinformatics and Systems Biology, National Yang Ming Chiao Tung University, Hsinchu, Taiwan

[†]These authors contributed equally to this work

[*]To whom correspondence should be addressed: JJ Lu: janglu45@gmail.com, JT Horng: horng@db.csie.ncu.edu.tw and TY Lee: leetzongyi@cuhk.edu.cn

Supplementary Materials

Machine Learning Models

There are numerous machine learning methods can be implied for prediction. Previous studies analyzed mass spectra used decision tree (DT)[1], random forest (RF)[2, 3], support vector machine (SVM)[1, 2, 4-7], neural network (NN)[6, 8] and k-nearest neighbor (KNN)[1, 2]. In our study, after clustering, we adopted four machine learning methods, logistic regression (LR), SVM, RF and extreme gradient boosting (XGBoost), to build up classification models for identification of the resistance of *E. coli* isolates through 5-fold cross validation (CV) with the default parameters provided by python. Then, we chose the model which attained the highest area under the receiver operating characteristic curve (AUC) built by XGBoost, and tuned the hyperparameters to the obtain higher AUC in 5-fold CV.

LR is a statistical model which is widely used in statistics. It basically uses a logistic function to estimate a binary dependent variable. It estimates the parameters of binary logistic model, in this case, resistant and not resistant represented by 1 and 0. The estimation or called prediction is the log-odds of the value labeled "1" which is calculated by a linear combination of multiple variables, also called features or predictors. Then log-odds are converted by the logistic function. The general formula can be expressed as follow:

$$F(y) = \frac{1}{1+e^{-y}}, \text{ where } y = a_0 + \sum_{i=1}^{n} a_i x_i.$$

F(*y*) is the predictive value of model and the formula is logistic function to convert log-odds of y, y is the linear combination of all predictors and a are the regression coefficients which are trained to minimize the difference between model prediction and ground truth. Note that logistic regression we used in our study was using *LogisticRegression* function in package scikit-learn *linear_model* in python [9]. Parameters were set by default. Several parameters worth mentioning are *penalty = 'l2'* which decided the l2 norm in the penalization, *tol = 0.0001* which set the tolerance of stopping criteria, *C = 1* which is the coefficient of regularization.

SVM is a supervised machine learning model which can use for classification and regression on linear or nonlinear data. What SVM do is separating the data by a hyperplane and extending the boundaries margin by the kernel trick. In another words, SVM algorithm will separate the data by searching an optimal linear hyperplane as wide

as possible. If data are non-linear classification, SVM also can perform kernel trick which maps isolates into high dimension space. The general SVM formula is as follow:

$$y_i\left(W^T x_i + b\right) \geq 1,\ \forall i$$

where *W* is the boundary hyperplane normal vector and the cost function of SVM is

$$\frac{W^T W}{2}.$$

We used *SVC* function in scikit-learn *svm* package in python [9] in this study. The SVM parameters were set by default. Some important parameters are *kernel = 'rbf'* which uses RBF kernel function to map the data, *C = 1* is the coefficient of penalty term, *probability = True* which enforce model prediction output probability.

RF is an ensemble learning method that fits multiple DT for classification. RF combines numerous DT's results based on majority vote and each DT will be trained by part of data or part of features. It can properly prevent the overfitting and usually yields the higher accuracy than DT. DT is a tree structure like classification method. The internal node of tree represents a condition of feature to separate the data. Following the flowchart of tree, finally each leaf of tree represents the class label. The basic idea of which feature to choose to split is the information gain, in other words, the entropy difference before and after splitting. The criterion of RF we used in this study is Gini index which calculates the impurity of each partition. The Gini index formula is:

$$Gini(D) = 1 - \sum_{i=1}^{m} p_i^2$$

where D is the isolates which dataset contains from n classes. And p is the probability of each class. Suppose data is split on A into subset, the gini index given the split on A is:

$$Gini_A(D) = \sum_i \frac{D_i}{D} Gini(D_i),$$

then the reduction of impurity is $\Delta Gini(A) = Gini(D) - Gini_A(D)$. Choosing the maximum *Gini(D)-Gini$_A$(D)* attribute to split the node which means choosing the largest reduction in impurity to make splitting data most separate. In our study, we used scikit-learn *ensemble* package *RandomForestClassifier* in python [9]. The *n_estimators* which indicates the number of decision trees we build in RF we chose 200. Other RF parameters were set by default. Some worth mentioning parameters we show as follow: *criterion = 'gini'* which decides using gini index to measure the splitting quality,

max_depth which forbids the tree depth splitting over this threshold, *min_sample_split = 2* which means the minimum number the internal nodes should split, *min_samples_leaf = 1* which sets the minimum number of forbidding splitting when leaf samples under this threshold.

XGBoost is an efficient, flexible, and portable supervised machine learning algorithms under the gradient boosting framework. It was implements by Chan, T., et al. [10]. XGBoost is DT ensembles based on classification and regression trees (CART). XGBoost training optimizes the objection function which contains training loss and regularization. It makes model training in a predictive, simple, and stable way. XGBoost also supports the parallel tree boosting making it training faster. Tree boosting is the method XGBoost chose except learn all the trees at once. It uses additive training strategy that trains and adds one new tree at one time. In the general additive training, the formula of prediction value at step t is as follow:

$$\hat{y}_i^{(t)} = \sum_{k=1}^{t} f_k(X_i) = \hat{y}_i^{(t-1)} + f_t(x_i).$$

Also, we need the objective function to minimize and train. In the general case, XGBoost takes the Taylor expansion of the loss function up to the second order, and the loss of interest takes logistic loss. Then the objective function becomes:

$$obj^{(t)} = \sum_{i=1}^{n} \left[l\left(y_i, \hat{y}_i^{(t-1)}\right) + g_i f_t(x_i) + \frac{1}{2} h_i f_t^2(x_i) \right] + \Omega(f_t) + const.,$$

where l represents logic loss function and the g_i and h_i are defined as:

$$g_i = \partial_{\hat{y}_i^{(t-1)}} l\left(y_i, \hat{y}_i^{(t-1)}\right)$$

$$h_i = \partial^2_{\hat{y}_i^{(t-1)}} l\left(y_i, \hat{y}_i^{(t-1)}\right)$$

and the last we need to explain is the regularization term:

$$\Omega(f) = \gamma T + \frac{1}{2} \lambda \sum_{j=1}^{T} w_j^2$$

where w is the vector of scores on leaves, T is the number of leaves. γ and λ are the hyperparameter we can tune while training. In our study, we used the *XGBClassifier* function in the *xgboost* package provided in python [10]. Also, we implemented the *GridSearchCV* function to tune the model which is provided in *grid_search* function in scikit-learn package [9]. Before tuning, we set parameter as follow: *eta = 0.1, max_depth = 10, gamma = 0.01, subsample = 0.8, colsample_by_tree = 0.9*. We tuned the model for four stages apart not just tuned it at a same time due to the time complexity. Each stage of model tuning was in grid search and 5 CV. First, we tuned model in setting

parameter *max_depth* and *min_child_weight* from range 9 to 13 and range 1 to 5 with increasing one at one time. That means we built 25 models at this stage. In second stage, we tuned model for parameter *gamma* from 0 to 0.4 with increasing 0.1 at one time. Then in third stage, we tuned model for *subsample* and *colsample_bytree* both from 0.75 to 0.95 with increasing 0.05 at one time. In the fourth stage, we tuned mode for *reg_alpha* in 0.0001, 0.001, 0.01, 0.1 and 0. Each stage was tuned in 0.1 *learning_rate*, 140 *n_estimator*, seed = 27 and *scale_pos_weight* = the ratio of susceptible to resistant. Also, the evaluation of *scoring* was set *roc_auc*. Finally, we got the best hyperparameters for CIP resistance prediction. Then we built the model by all training data in the best tuned parameters with *learning_rate* = 0.001, *n_estimator* = 2000, and it would be the finally models for our study to test the independent test.

Supplementary Figures

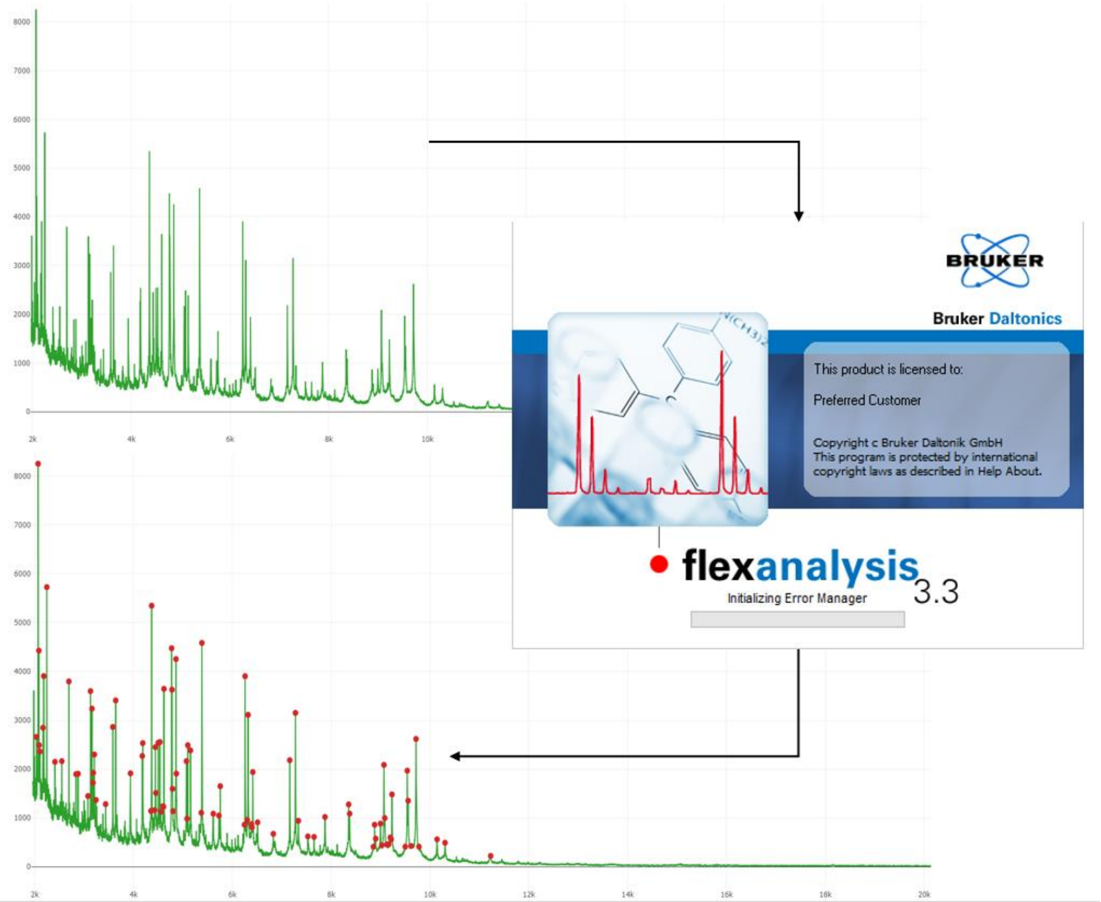

Supplementary Figure S1. Processing of MS spectrum by flexAnalysis.

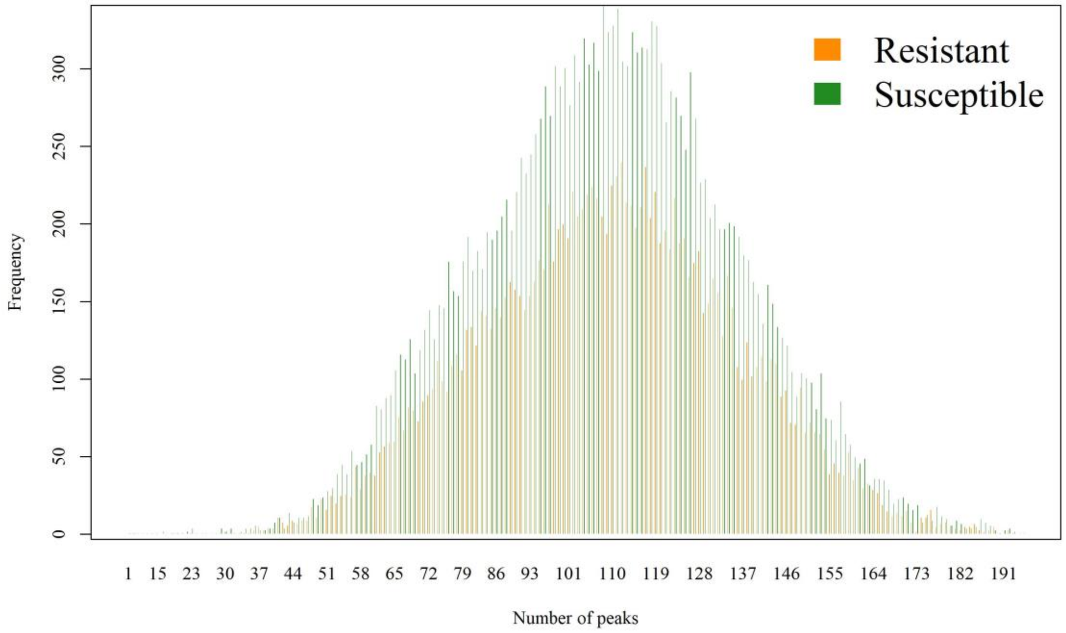

Supplementary Figure S2. Spectral peaks number of the *E. coli* isolates. Note: x label is the count peaks of each isolate, y label is the number of isolates, orange bar represents the CIP resistant isolates, and green bar represents the CIP susceptible isolates.

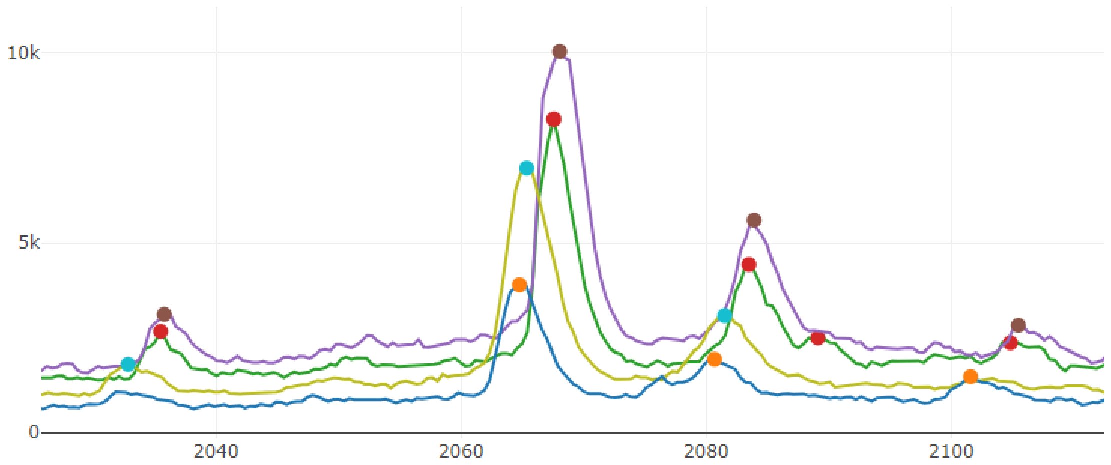

Supplementary Figure S3. Shifting problem of MALDI-TOF MS spectra.

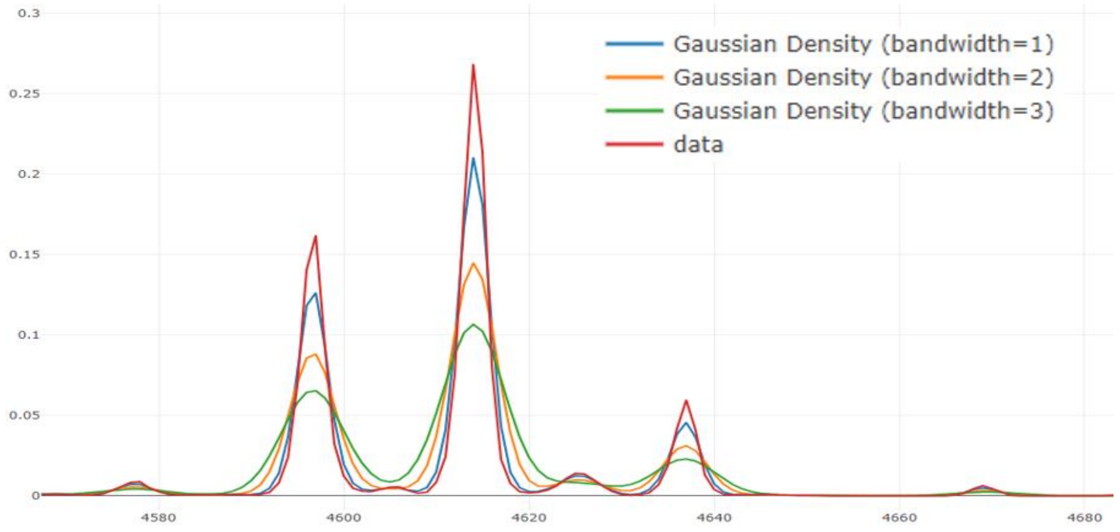

Supplementary Figure S4. Gaussian kernel density with different bandwidth.

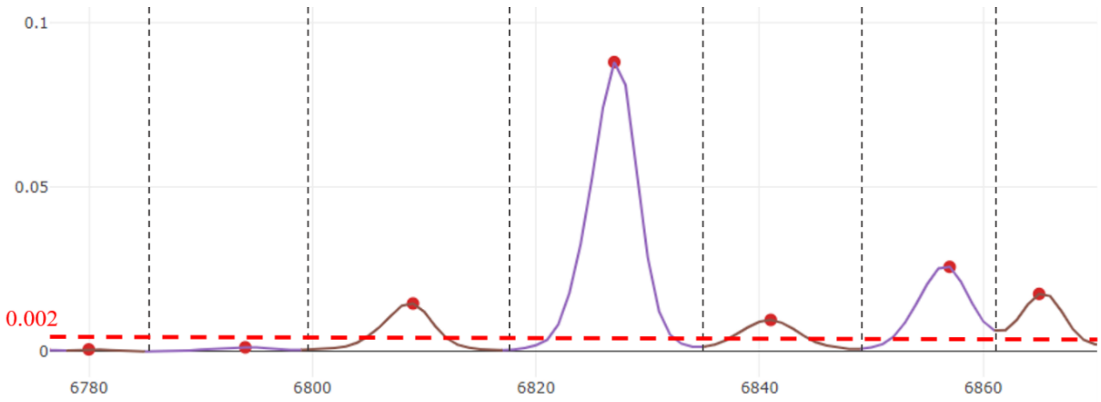

Supplementary Figure S5. Peak detection and alignment of Gaussian kernel density.

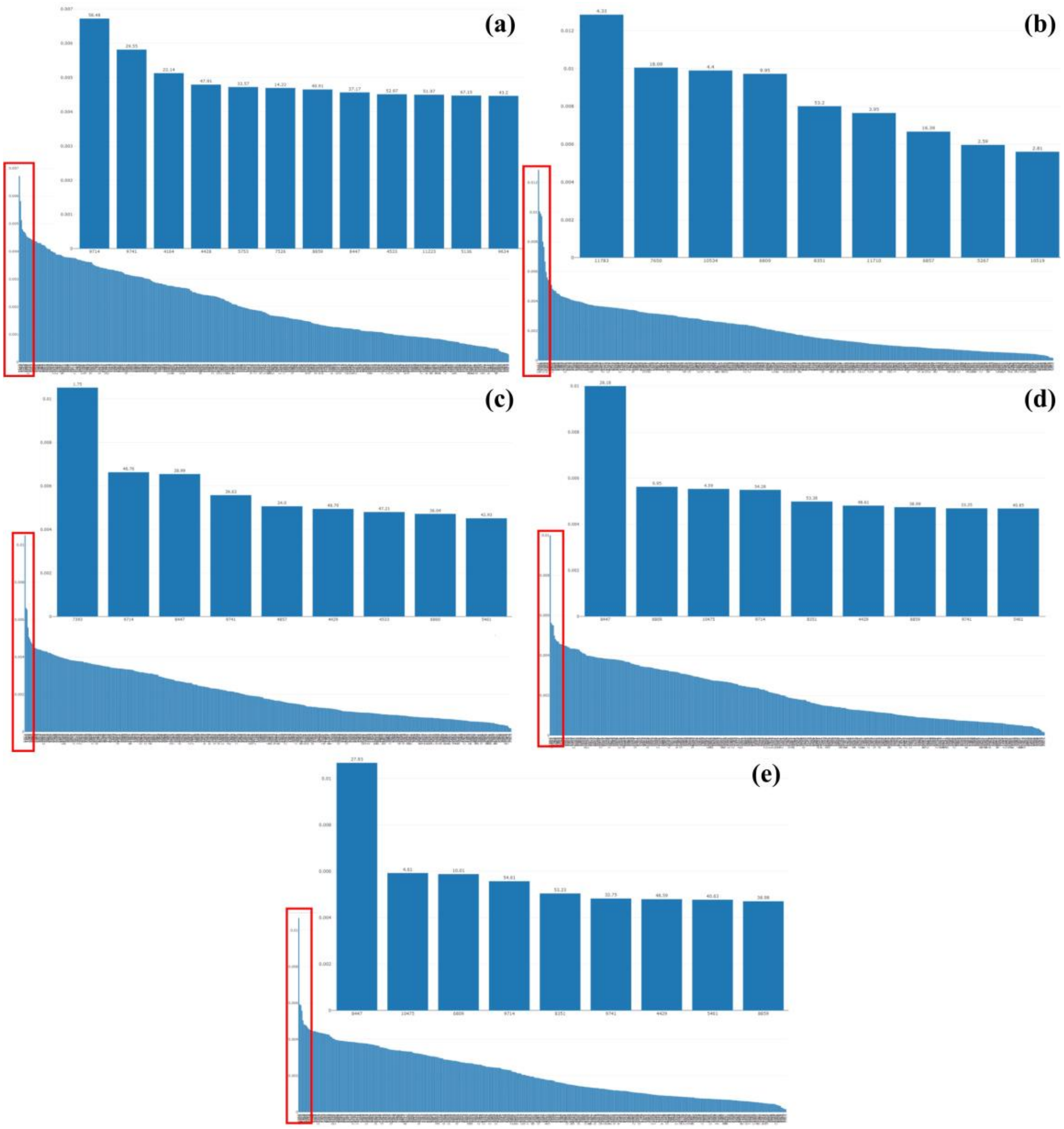

Supplementary Figure S6. The feature importance order for detecting resistance to (a) AMC, (b) CIP, (c) CAZ, (d) CRO, and (e) CXM based on random forest classifier.

Supplementary Tables

Supplementary Table S1. Numbers of data and susceptible proportion for each antibiotic.

Antibiotic	All_RSI	All_RS	S%	All_RSI	All_RS	S%
	Linkou (n=38,595)			Kaohsiung (n=9,628)		
**AMC**	22,695	20,132	83.0%	5,592	5,235	83.9%
**CIP**	37,619	37,079	59.0%	9,364	9,281	57.2%
**CAZ**	13,832	12,561	71.2%	3,775	3,551	77.8%
**CRO**	37,640	37,072	64.3%	9,368	9,210	68.2%
**CXM**	37,619	36,352	63.5%	9,368	9,119	66.7%

Supplementary Table S2. Statistical data of each sample's peaks in Linkou dataset.

**Linkou**	**Mean**	**Std**	**Min**	**25%**	**50%**	**75%**	**Max**
(n=38,595)	114.39	28.49	1	95	114	133	200

Supplementary Table S3. 5-fold CV random forest average results of all dataset and dataset with informative peak presence.

Antibiotic		ACC	SEN	SPC	AUC
AMC	ALL	63.24	59.41	64.03	0.652
	Samples with 4533 presence	63.14	59.14	63.91	0.654
CIP	ALL	68.55	66.62	69.9	0.764
	Samples with 9714 presence	74.13	72.63	75.34	0.83
CAZ	ALL	61.97	58.26	63.43	0.659
	Samples with 9714 presence	63.85	61.35	64.6	0.673
CRO	ALL	63.62	61.05	65.04	0.69
	Samples with 9714 presence	67.78	66.1	68.56	0.736
CXM	ALL	63.97	61.33	65.5	0.695
	Samples with 9714 presence	68.01	65.88	69.04	0.738

Supplementary Table S4. Performance of informative peak absence models on 5-fold cross validation. The informative peak for AMC is *m/z* 4533 and other four antibiotics are *m/z* 9714.

		Machine learning algorithms			
**Antibiotics**	**Metrics**	**LR**	**RF**	**SVM**	**XGB**
**AMC**	**ACC (%)**	59.65±1.04	62.62±1.58	59.12±1.44	60.50±1.39
	**SEN (%)**	59.59±0.90	59.80±1.45	58.86±1.45	60.31±1.17
	**SPE (%)**	59.66±1.08	63.27±1.60	59.18±1.44	60.54±1.45
	**AUC**	0.634±0.01	0.654±0.02	0.623±0.016	0.651±0.012
**CAZ**	**ACC (%)**	57.98±0.50	57.64±1.00	58.46±0.73	58.05±0.85
	**SEN (%)**	57.83±0.58	53.48±1.80	58.41±0.68	57.88±0.93
	**SPE (%)**	58.05±0.47	59.73±0.94	58.50±0.76	58.14±0.82
	**AUC**	0.612±0.005	0.597±0.012	0.622±0.009	0.613±0.01
**CIP**	**ACC (%)**	60.14±1.01	61.34±0.91	60.56±0.74	61.39±0.65
	**SEN (%)**	60.10±1.02	58.36±1.21	60.51±0.75	61.33±0.66
	**SPE (%)**	60.16±1.00	63.05±1.27	60.59±0.74	61.42±0.65
	**AUC**	0.651±0.01	0.658±0.006	0.653±0.007	0.668±0.009
**CRO**	**ACC (%)**	57.79±0.67	58.01±1.13	58.29±0.75	58.04±1.11
	**SEN (%)**	57.73±0.66	54.45±0.87	58.25±0.75	57.98±1.10
	**SPE (%)**	57.82±0.67	60.42±1.35	58.32±0.74	58.07±1.12
	**AUC**	0.612±0.007	0.605±0.015	0.616±0.006	0.616±0.011
**CXM**	**ACC (%)**	57.95±0.41	57.64±0.94	58.38±0.91	57.73±1.50
	**SEN (%)**	57.93±0.46	54.34±1.65	58.29±0.89	57.68±1.52
	**SPE (%)**	57.97±0.38	60.00±0.66	58.44±0.92	57.76±1.49
	**AUC**	0.615±0.007	0.61±0.009	0.62±0.005	0.615±0.017

Note: AMC: amoxicillin; CAZ: ceftazidime; CIP: ciprofloxacin; CRO: ceftriaxone; CXM: cefuroxime; ACC: accuracy; SEN: sensitivity; SPE: specificity; AUC: area under the receiver operating characteristics curve; LR: logistic regression; RF: random forest; SVM: support vector machine; XGB: extreme gradient boosting.

References

1. Wang HY, Lee TY, Tseng YJ et al. A new scheme for strain typing of methicillin-resistant Staphylococcus aureus on the basis of matrix-assisted laser desorption ionization time-of-flight mass spectrometry by using machine learning approach, Plos One 2018;13:e0194289.
2. De Bruyne K, Slabbinck B, Waegeman W et al. Bacterial species identification from MALDI-TOF mass spectra through data analysis and machine learning, Systematic and applied microbiology 2011;34:20-29.
3. Gu M, Buckley M. Semi-supervised machine learning for automated species identification by collagen peptide mass fingerprinting, BMC Bioinformatics 2018;19:241.
4. Salman A, Sharaha U, Rodriguez-Diaz E et al. Detection of antibiotic resistant Escherichia Coli bacteria using infrared microscopy and advanced multivariate analysis, Analyst 2017;142:2136-2144.
5. Saeed F, Pisitkun T, Knepper MA et al. An efficient algorithm for clustering of large-scale mass spectrometry data. In: 2012 IEEE International Conference on Bioinformatics and Biomedicine. 2012, p. 1-4. IEEE.
6. Skarysz A, Alkhalifah Y, Darnley K et al. Convolutional neural networks for automated targeted analysis of raw gas chromatography-mass spectrometry data. In: 2018 International Joint Conference on Neural Networks (IJCNN). 2018, p. 1-8. IEEE.
7. Lee J, Shin Y, Kim S et al. SVM Classification Model of Similar Bacteria Species using Negative Marker: Based on Matrix-Assisted Laser Desorption/Ionization Time-of-Flight Mass Spectrometry. In: 2017 IEEE 17th International Conference on Bioinformatics and Bioengineering (BIBE). 2017, p. 145-150. IEEE.
8. Rumelhart DE, Hinton C, Williams R. MSnet: A Neural Network That Classifies Mass Spectra 1990.
9. Pedregosa F, Varoquaux G, Gramfort A et al. Scikit-learn: Machine Learning in Python, Journal of Machine Learning Research 2011;12:2825-2830.
10. Chen T, Guestrin C. Xgboost: A scalable tree boosting system. In: Proceedings of the 22nd acm sigkdd international conference on knowledge discovery and data mining. 2016, p. 785-794. ACM.
